# Spectrofluorometric determination of orphenadrine, dimenhydrinate, and cinnarizine using direct and synchronous techniques with greenness assessment

**DOI:** 10.1038/s41598-023-40559-x

**Published:** 2023-08-20

**Authors:** Rana Ghonim, Manar M. Tolba, Fawzia Ibrahim, Mohamed I. El-Awady

**Affiliations:** 1 Department of Pharmaceutical Analytical Chemistry, Faculty of Pharmacy, Mansoura University, Mansoura, 35516 Egypt; 2 Department of Pharmaceutical Chemistry, Faculty of Pharmacy, Delta University for Science and Technology, International Coastal Road, Gamasa, 11152 Egypt

**Keywords:** Analytical chemistry, Green chemistry

## Abstract

Orphenadrine (ORP), dimenhydrinate (DMN), and cinnarizine (CNN) were investigated using green-sensitive spectrofluorometric methods. Method, I used for determination of DMN in 0.1 M hydrochloric acid (HCl) and 1.0% sodium dodecyl sulphate (SDS) at 286 nm after λ_ex_ 222 nm, while for determination of ORP in 1.0% w/v SDS involves measuring the fluorescence at 285 nm after λ_ex_ 220 nm. For DMN and ORP, the detection and quantitation limits were 2.99 and 4.71 and 9.08 and 14.29 ng/mL, respectively. The ranges of DMN and ORP were 0.10–1.0 and 0.04–0.5 µg/mL, respectively, in micellar aqueous solution. Method II, the derivative intensities of DMN and CNN were measured at a fixed of different wavelength between the excitation and the emission wavelengths (Δλ) = 60 nm at 282 and 322 nm, at the zero crossing of each other, respectively. The detection and quantitation limits for DMN and CNN were 1.77 and 0.88 ng/mL and 5.36 and 2.65 ng/mL, correspondingly, through the entire range of 0.1–1.0 µg/mL for DMN and CNN. The linearity was perfectly determined through the higher values of the correlation coefficient ranging from 0.9997 to 0.9999 for both direct and synchronous methods. The precision of the proposed methods was also confirmed via the lower values of the standard deviation which ranged from 0.39 to 1.11. The technique was expanded to analyze this mixture in combined tablets and laboratory-prepared mixtures. The method validation was done depending on the international conference on harmonization (ICH) recommendations. An analysis of the statistical data revealed a high agreement between the proposed data and the comparison methodology. Three different assessment methods demonstrated the greenness of the technique.

## Introduction

**Figure 1:**
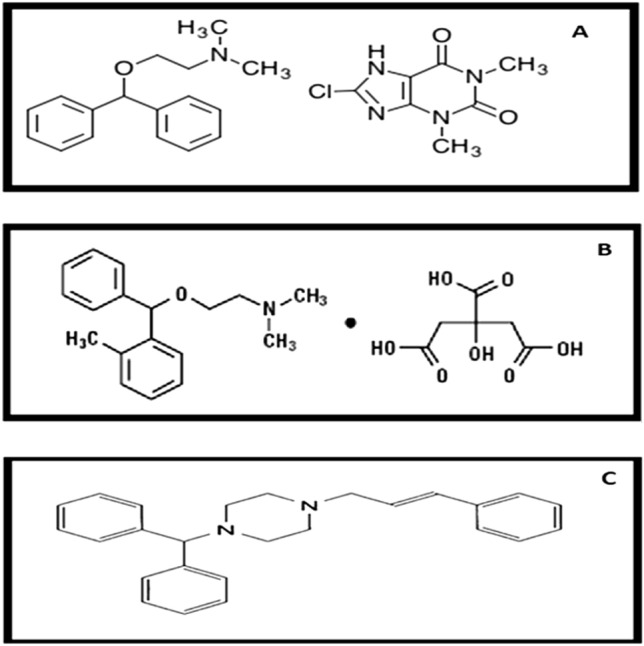
Chemical formulae of: (**A**) dimenhydrinate (DMN). (**B**) orphenadrine citrate (ORP). (**C**) cinnarizine (CNN).

Dimenhydrinate (DMN; Fig. 1A) is classified as a mixture of 2-(diphenyl methoxy)-N,N-dimethylethanolamine and 8-chloro-3,7-dihydro-1,3-dimethyl-1H-purine-2,6-dione^1^. N,N-dimethyl-2-[(2-methyl phenyl) phenyl methoxy] ethanamine is orphenadrine citrate (ORP; Fig. 1B)^1^. The chemical name for cinnarizine (CNN; Fig. 1C) is (E)-1-(diphenylmethyl)-4-(3-phenyl prop-2-enyl)piperazine^2^. DMN, ORP, and CNN have been recognized as medications in British Pharmacopeia (BP)^2^ and United States Pharmacopeia (USP)^3^. DMN and CNN are antihistaminic drugs with sedative and antimuscarinic properties. They are mainly utilized as an antiemetic to treat and prevent motion sickness. Additionally, they treat the symptoms of vertigo and nausea brought on by Meniere’s disease and other vestibular abnormalities^4^. In the tablet dosage forms like (Arlevert^®^ and Cizinate^®^), CNN and DMN are combined in a pharmaceutical ratio of 1:2 w/w. For more than three decades, the fixed combination of cinnarizine 20 mg and dimenhydrinate 40 mg has been used to treat vertigo for various reasons. The dual mechanism of action is due to the calcium channel blocker cinnarizine, which mostly affects the peripheral vestibular system, and dimenhydrinate, which largely affects the central vestibular system^5^.

Due to the overlapping between the excitation and emission spectra of DMN and CNN, specific selectivity issues could arise, especially in multi-drug analyses. This issue was resolved by synchronous fluorescence spectroscopy (SFS). Our technique is a form of SFS known as constant wavelength synchronous fluorescence spectroscopy, which employs a constant difference between wavelengths (CWSFS). As a result, SFS has a significant advantage over traditional fluorescence, boosting spectral resolving and light divergence. The SFS method and derivative amplitude work together to give excellent resolution for both medications^6^.

According to the literature, some reports for DMN estimation, such as ultra violet (UV) spectrophotometry^7,8^. Both DMN and CNN were estimated by thin layer chromatography (TLC), RP-HPLC methods densitometric^9^. Other methods like voltametric techniques^10^, liquid chromatography (LC)—electrospray tandem mass spectrometry^11^, and other various techniques^12^. At the same time, versatile analytical techniques could be used for the estimation of ORP, like RP-HPLC^13,14^, derivative spectrophotometry^15–17^, liquid chromatography mass spectrometry/mass spectrometry (LC–MS/MS)^18^, and chemometric methods^19^. Different analytical techniques have been employed to determine CNN, such as reverse phase-high performance liquid chromatography (RP-HPLC)^20^, derivatization spectrophotometric technique^21–23^, spectrofluorimetry^24,25^, and voltametric technique^26^. Both DMN and CNN were estimated by thin layer chromatography (TLC), RP-HPLC methods^7,9^.

By surveying the literature, it was found that there were no previous reports concerning the conventional micellar fluorometric technique for the determination of DMN or ORP or synchronous fluorometric one for the assessment of a combined mixture of DMN and CNN.

The goal of this study is to use the standard conventional technique (Method I) to evaluate ORP or DMN alone and to simultaneously determine DMN in the presence of CNN by using first derivative synchronous fluorescence spectroscopy (FDSFS) as their co-formulation in a tablet dosage form (Method II). It is clear to detect a significant overlap between DMN and CNN while scanning their native fluorescence spectra. FDSFS is a well-known method for subjectively and quantitatively separating such a mixture (Method II). These two straightforward, extremely eco-friendly spectrofluorometric techniques are sensitive enough to quantify DMN, ORP, and CNN in commercial tablets and capsules. This work is exclusively reporting the first direct spectrofluorimetric determination (through native fluorescence) of dimenhydrinate and orphenadrine. In addition, the work includes, for the first time, the use of synchronous spectrofluorometric technique for the concurrent determination of dimenhydrinate and cinnarizine.

## Experimental

### Apparatus

Shimadzu RF-6000 spectrofluorophotometer with a 150 W Xenon flash lamp, high sensitivity mode, smoothing factor 10.00, slit width 5.00 nm, and 1.00 cm quartz cell were adjusted for the conventional spectrofluorometric measuring method. With scanning in the 200–600 nm range, synchronous spectrofluorometric measurements were carried out at Δλ = 60 nm. The Cary Eclipse software was used to gather the data that had been saved. The first derivative spectra were altered with a 19.00 filter size and a 1.00 nm spacing. The excitation and emission windows were 10 nm, and a 12,000 nm/min scan rate was chosen. pH was modified using a pH meter (Consort, NV P-901, Belgium). The sonicator was a Sonix IV type SS101H 230 from the USA. Electronic balance sartorius Entris 224-IS laboratory balance Model: 224-IS.

### Materials and solvents

EPICO supplied dimenhydrinate (DMN) and orphenadrine citrate (ORP) (the tenth of Ramadan in Cairo, Egypt). The Arab and pharmaceutical business El-Amereya, Cairo, Egypt, provided the cinnarizine.

The purity of DMN and CNN were certified to be 100.13% and 100.01%, respectively, checked by the comparison method^9^.

The purity of ORP was certified to be 100.19% checked by the comparison method^15^.

Dramanex^®^ tablets contain 50.0 mg DMN (batch # 11224), produced by Al-kahira, Shoubra, Egypt, and brought from a neighborhood pharmacy in Egypt.

Norflex^®^ ampoules each contain 30.0 mg/mL ORP (batch # 210697), produced by EPICO, 10th Ramadan, Cairo, Egypt.

Cinnarizine^®^-75 mg capsules (batch# 2170003), contain 75 mg CNN produced by the Arab and drug company El-Amereya, Cairo, Egypt, bought from a local pharmacy in Egypt.

To make DMN and CNN combined prepared tablets in their pharmaceutical ratio 2:1 w/w, the following ingredients were combined: 20.0 mg of DMN, 10.0 mg of CNN, 15.0 mg of lactose, 20.0 mg of talc powder, 15.0 mg of maize starch, and 10.0 mg magnesium stearate.

Analytical-grade chemicals and HPLC-grade solvents were used. Methanol, ethanol, acetonitrile, and n-propanol were purchased from Sigma-Aldrich (Germany) HPLC grade. At the same time, acetone was acquired in analytical grade from EL-Nasr Pharmaceutical Chemical Co. (ADWIC, Cairo, Egypt).

Surfactants like 94% sodium dodecyl sulfate (SDS), carboxy methyl cellulose (CMC), tween 80, cetrimide, and β-cyclodextrin (β-CD) were acquired from EL-Nasr Pharmaceutical Chemical Co. (ADWIC, Cairo, Egypt). They have been prepared into aqueous solutions containing 1.0% w/v.

Reagents like sodium hydroxide, boric acid, phosphoric acid, acetic acid, and hydrochloric acid were also used. Furthermore, they were bought from EL-Nasr Pharmaceutical Chemical Co. (ADWIC, Cairo, Egypt). For all procedures, de-ionized water was used throughout the entire process. Combining the appropriate volumes of 0.04 M phosphoric acid, 0.04 M boric acid, and 0.04 M acetic acid and correcting the pH using 0.2 M sodium hydroxide, Britton Robinson buffer with a pH range of 2.2–11.5 was created.

### Preparation of standard solution

DMN and CNN standard stock solutions were generated by dissolving 10 mg in a volumetric flask with 100 mL of ethanol, while 10 mg ORP was dissolved in 5 mL ethanol and then topped off to the mark with de-ionized water. The proper dilutions were then made. To achieve 10 µg/mL, the working solutions of the tested medicines were made in the appropriate solvent. Upon chilling, ORP remained constant for three days without change, whereas DMN and CNN needed to be freshly prepared^2^. Due to their photosensitivity, all medications should be protected from light by covering them with aluminum foil.

### Procedures

#### Methods for calibration graphs

##### Method I

Chosen volumes of the DMN working standard solution (10 µg/mL) were put on 10-mL volumetric flasks. The final concentration was in the linear range (0.10–1.0 µg/mL); therefore, 0.6 mL of the 0.1 M HCl and 1.80 mL of the 1.0% w/v SDS were added after that. The volume was then topped off with de-ionized water. At 222/286 nm, the fluorescence intensity was measured. The relative fluorescence intensity (RFI) was plotted against the relevant drug concentrations in µg/mL following the completion of the side-by-side blank experiment. The regression equation could then be derived.

Aliquots of ORP working standard solution were transferred into several 10-mL volumetric flasks, 1.0 mL of 1.0% w/v SDS was added, and the volume was topped off with de-ionized water for the ORP concentration to be within the linear extent (0.04–0.50 µg/mL). The fluorescence intensity was determined at 220/285 nm. After performing the side-by-side blank experiment, plots of the relative fluorescence intensity (RFI) and related drug concentrations in µg/mL were made. Then, the regression equation was created.

##### Method II

In several 10-mL volumetric flasks, aliquots of DMN and CNN working standard solutions encompassing the linear range of (0.1–1.0 µg/mL for DMN and CNN) were transferred. 1 mL of 1.0% w/v of SDS was added and diluted to the mark with de-ionized water. Then the solutions’ SFS were captured at a constant wavelength difference of Δλ of 60 nm. Then, the first derivative synchronous fluorescence spectra (FDSFS) of DMN and CNN were generated. For DMN and CNN, the peak amplitudes of the first derivative spectra (^1^D) were measured at 282 nm and 322 nm, respectively. For any measurement errors, parallel blank experiments were conducted. The calibration graph and accompanying regression equations were then created by plotting the peak amplitude of the ^1^D spectra against the drug concentration in µg/mL.

#### Validation of analytical procedures

Following ICH Q2 (R1) guidelines^27^, typical validation characteristics were investigated adopting the following procedures:

##### Linearity and range

The linearity was evaluated by visual inspection of a plot of signals as a function of analyte concentration. The linear relationship was evaluated by regression analysis. The correlation coefficient, y-intercept, slope of the regression line and residual sum of squares are calculated. In addition, an analysis of the deviation of the actual data points from the regression line was also calculated. As recommended by ICH, a minimum of 5 concentrations is investigated.

The range was derived from linearity studies. It was established by confirming that the analytical procedure provides an acceptable degree of linearity, accuracy and precision when applied to samples containing amounts of analyte within or at the extremes of the specified range of the analytical procedure. For the assay of a drug substance or a finished product, the range is normally from 80 to 120% of the test concentration.

##### Accuracy

Accuracy was established across the specified range of the analytical procedure by comparing the results of the proposed analytical procedure with those of a second well-characterized procedure, the accuracy of which is stated. Recommended data for accuracy should be assessed using a minimum of 9 determinations over a minimum of 3 concentration levels covering the specified range (3 concentrations/3 replicates each of the total analytical procedure). Accuracy was reported as percent recovery by the assay of known added amount of analyte in the sample.

##### Limit of detection (LOD) and limit of quantitation (LOQ)

LOD is the lowest concentration that could be detected and calculated at 3.3 S_a_/b, while LOQ is the lowest concentration that could be quantified in terms of accuracy and precision and calculated at 10 S_a_/b.; where S_a_ means that the standard deviation of the intercept of the regression line is b, the slope of the calibration graph.

##### Precision

The assessment of precision includes intra-day and inter-day precisions using a minimum of 9 determinations covering the specified range for the procedure (3 concentrations/3 replicates each). Recommended data for precision includes the standard deviation and relative standard deviation (coefficient of variation).

##### Robustness

The robustness was verified by assessing the effect of small deliberate changes in different experimental parameters including the variations in pH (1.3 ± 0.2) and volume of the acid (0.6 mL ± 0.2 mL) and surfactant 1% w/v SDS (1.8 mL ± 0.2 mL) for DMN, (1 mL ± 0.2 mL) for ORP in Method I and 1% w/v SDS w/v (1 mL ± 0.2 mL) for DMN and CNN in Method II.

##### Selectivity

The selectivity was evaluated by testing for excipient interference in the pharmaceutical formulations using both methods including talc, magnesium stearate, or lactose.

#### Analysis of DMN/ CNN synthetic mixtures

From their typical stock solutions, synthetic mixtures of CNN and DMN in the concentration range shown in Table S1 were produced in various ratios. The mixtures were handled following "Methods for calibration graphs" section. (Method II). The peak amplitudes of the ^1^D synchronous spectra and the percent recoveries were calculated concurrently for each drug using the constructed calibration graphs or the corresponding regression equations.

#### Analysis of pharmaceutical preparations

##### Single dosage forms; tablets, capsules, and ampoules

Ten Dramanex^®^ tablets were triturated and well blended. To achieve a 100.0 µg/mL analyte, an equivalent amount to 10.0 mg analyte was placed into a 100 mL volumetric flask and extracted by ethanol. After 30 min of sonication, the solution was filtered.

The contents of Five Norflex^®^ ampoules containing ORP were thoroughly combined. The solution was adequately measured and poured into 100 mL volumetric flasks before being finished with de-ionized water.

The working solutions (0.10–1.0 µg/mL for DMN and 0.04–0.50 µg/mL for ORP) were then prepared by dilution with water. As mentioned above, spectrofluorimetric assay experiments (Method I) and calculations of percentage recoveries were finally performed.

The contents of ten cinnarizine^®^ capsules were mixed well. Then an amount of powdered analyte ≡ 10 mg was weighed, added to 100 mL volumetric flasks, and topped off to the mark with ethanol. Sonication was applied for half an hour, and then samples were filtered. The working solutions (0.10–1.0 µg/mL) were diluted with water. As mentioned above, spectrofluorimetric assay experiments (Method II) and calculations of percentage recoveries were finally performed.

##### Co-formulated tablets

Ten in-lab-prepared tablets with a medicinal ratio of 1:2 w/w for CNN and DMN were weighed, thoroughly combined, finely ground and then compressed. In a 100 mL volumetric flask, a portion of the powder equal to 10.0 mg of CNN and 20.0 mg of DMN was placed. About 80 mL of ethanol was transferred, and the solutions were sonicated for 30 min, topped off with the same solvent, and filtered. The previously described process was followed. As shown above, the first derivative synchronous fluorescence spectroscopy (FDSFS) analysis was carried out (Method II). The amounts of each medicine in the co-formulated tablets were calculated using regression equations specific to each drug.

## Results and discussion

**Figure 2:**
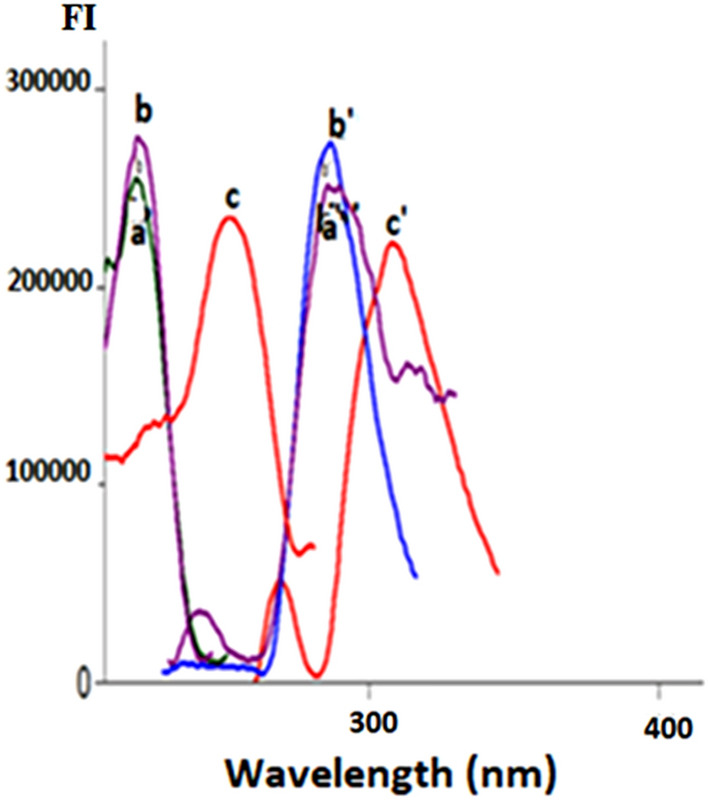
The excitation and emission spectra of ethanolic solution of 0.4 µg/mL: a, b, c are the excitation spectra of dimenhydrinate (DMN), orphenadrine (ORP), cinnarizine (CNN). While a**′**, b′, c′ are the emission spectra of dimenhydrinate (DMN), orphenadrine (ORP), cinnarizine (CNN).

**Figure 3:**
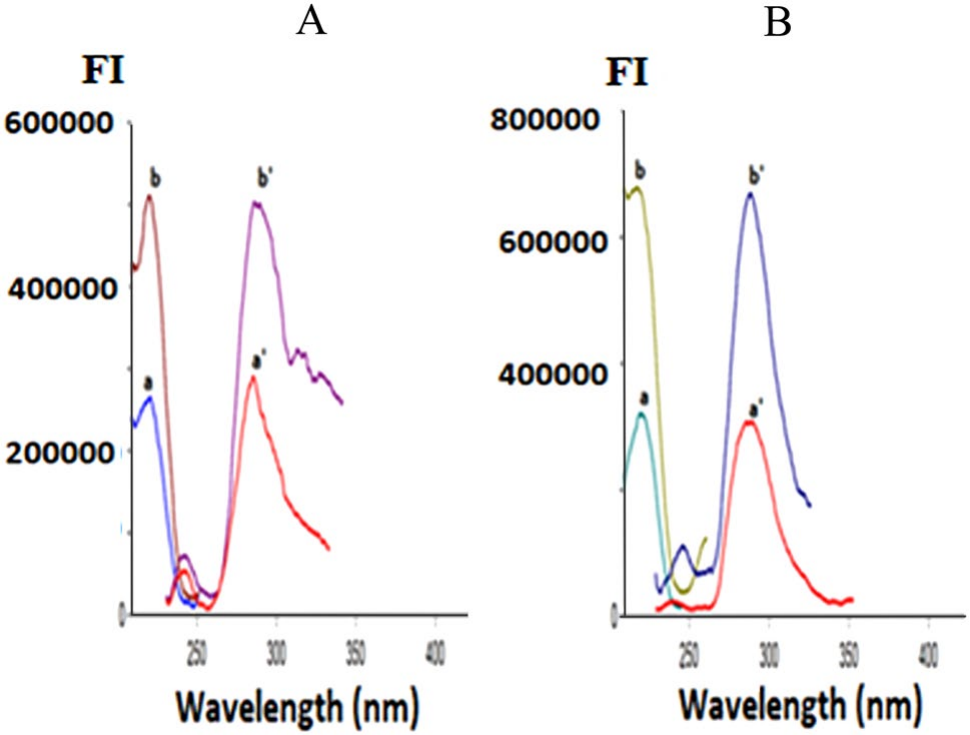
The fluorescence spectra of 0.40 µg/mL of: (**A)** a, a′ Aqueous acidic solution of dimenhydrinate DMN. b, b′ Aqueous acidic micellar solution of DMN. (**B**) a, a′ Aqueous solution of orphenadrine citrate ORP. b, b′ Aqueous micellar solution of ORP.

DMN, ORP, or CNN all exhibit native fluorescence in their ethanolic solutions at wavelengths of 222/286 nm, 220/285 nm, and 250/308 nm, correspondingly, as presented in (Fig. 2). As shown in (Fig. 3), the addition of 1% SDS w/v significantly improved the emission spectra of 0.4 µg/mL DMN in an aqueous acidic solution at 286 nm and 0.4 g/mL ORP in its aqueous solution at 285 nm. As a result, a new accurate, precise conventional spectrofluorimetric method was proposed to directly determine the two analytes in bulk powder and pharmaceutical formulations (Method I).

A significant overlap was observed between DMN and CNN's emission spectra, which conventional spectrofluorometry could not separate (Fig. 2).

**Figure 4:**
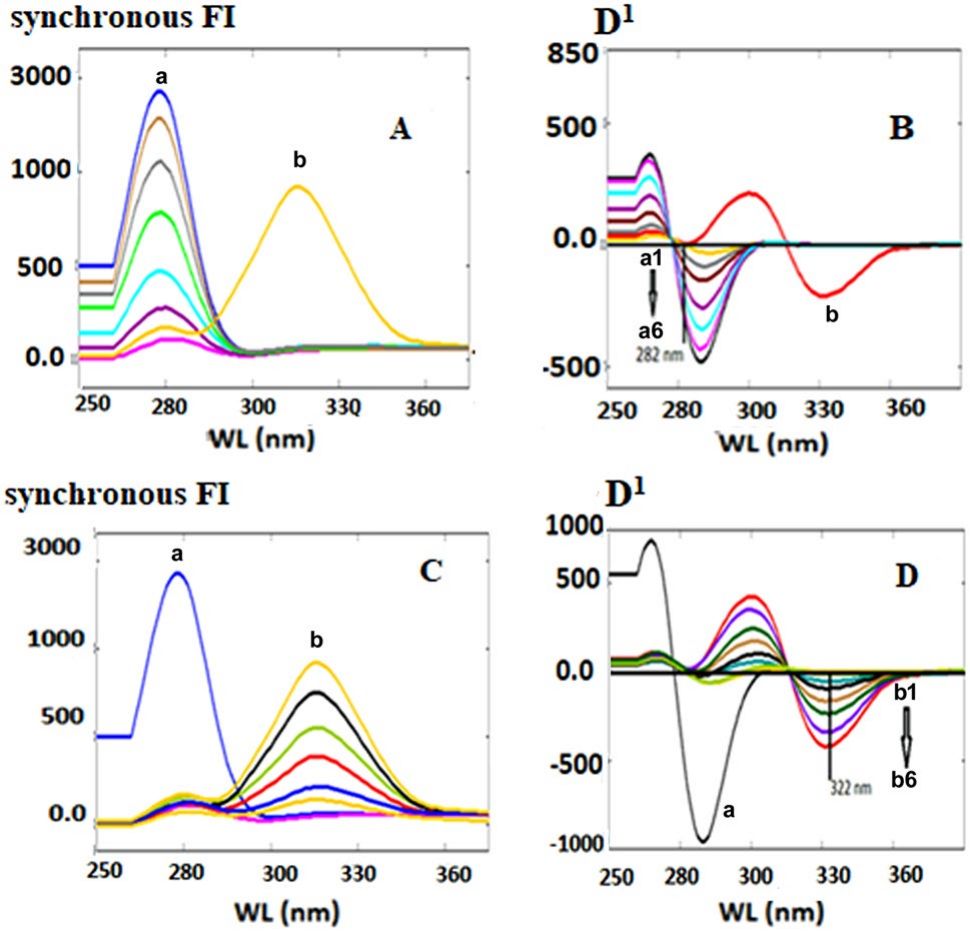
Different concentrations of DMN and CNN using SFS and FDSFS conditions in which: (**A**) is SFS conditions (a is different concentrations of DMN starts from 0.1 to 1.0 µg/mL and b is 1.0 µg/mL CNN). (**B**) is FDSFS conditions (a1: a6 is different conditions of DMN starts from 0.1 to 1.0 µg/mL at 282 nm and b is 1.0 µg/mL CNN). (**C**) is SFS conditions (a is 1.0 µg/mL DMN and b is different concentrations of CNN starts from 0.1 to 1.0 µg/mL). (**D**) is FDSFS conditions (a is 1.0 µg/mL DMN and from b1: b6 is different concentrations of CNN starts from 0.1 to 1.0 µg/mL at 322 nm).

The SFS of DMN and CNN was scanned at numerous Δλ (20–200 nm) to select the optimum Δλ at which both analytes exhibit high sensitivity and selectivity. Figure 4A,C shows that DMN and CNN synchronous fluorescence spectra overlapped, as the luminescence spectra of CNN greatly interfere with that of DMN. Consequently, it isn't easy to quantify and separate them simultaneously; Due to that, the SFS of the different concentrations of CNN does not read zero at the maxima of DMN; So, the first derivative SFS was adopted to estimate the two drugs concurrently. DMN could be determined by FDSFS at 282 nm at the zero-crossing point of CNN, while CNN could be well quantitated at 322 nm at the zero-crossing point of DMN, as shown in (Fig. 4B and D).

### Optimizing the experimental conditions

Studying factors affecting sensitivity and selectivity refined the approach. These parameters included solvents, pH, surfactants, etc. The proposed procedures were validated to assay DMN, ORP, and CNN in bulk and pharmaceutical dosage forms.

#### Effect of diluting solvents

Six solvents were examined to find the best one for fluorometric pharmaceutical analysis with the maximum luminescence intensity.

**Figure 5:**
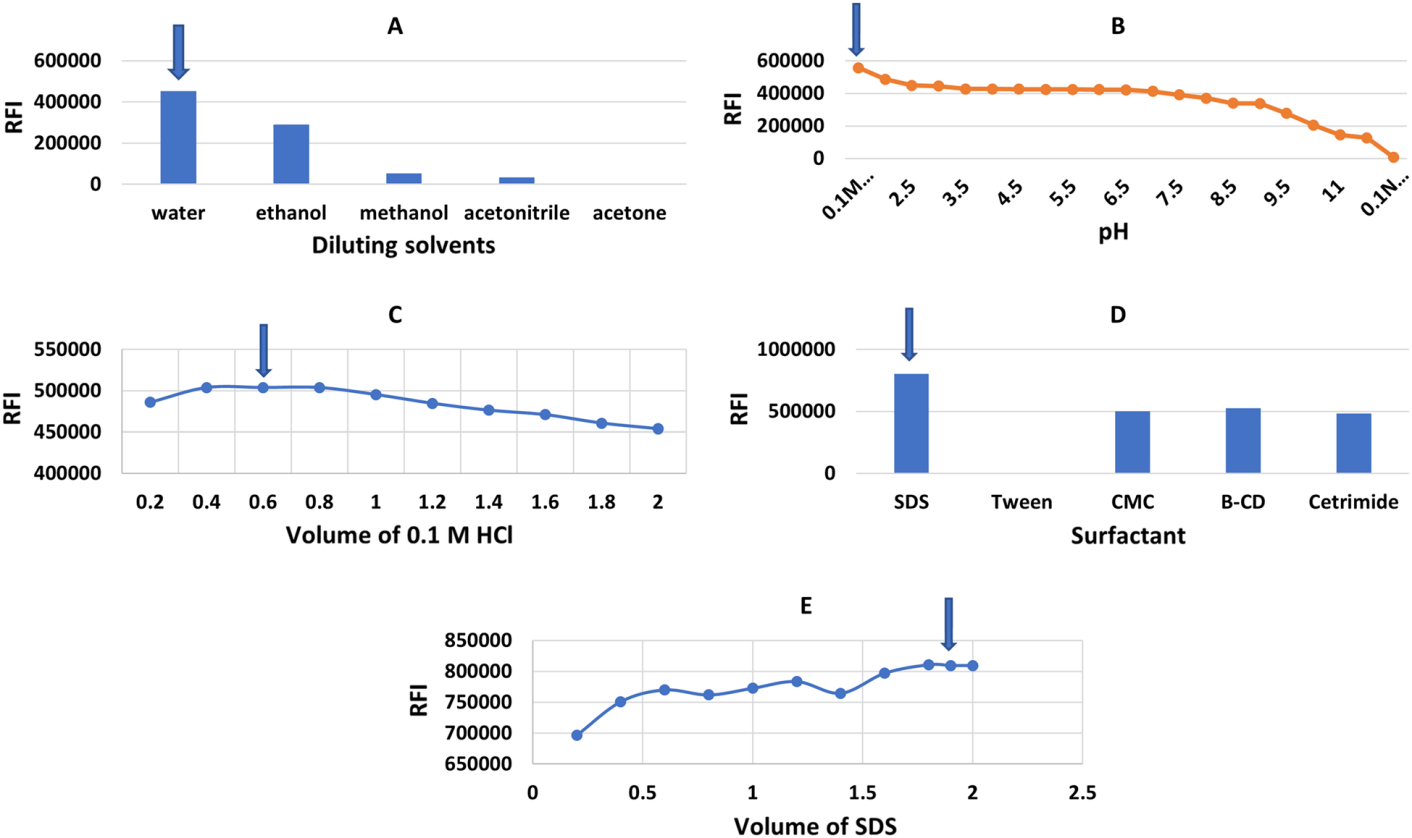
Optimization of the experimental conditions for determination of DMN: (**A**) effect of diluting solvent. (**B**) effect of pH. (**C**) effect of volume of 0.1N HCl. (**D**) effect of surfactants. (**E**) effect of volume of 1.0% w/v SDS.

**Figure 6:**
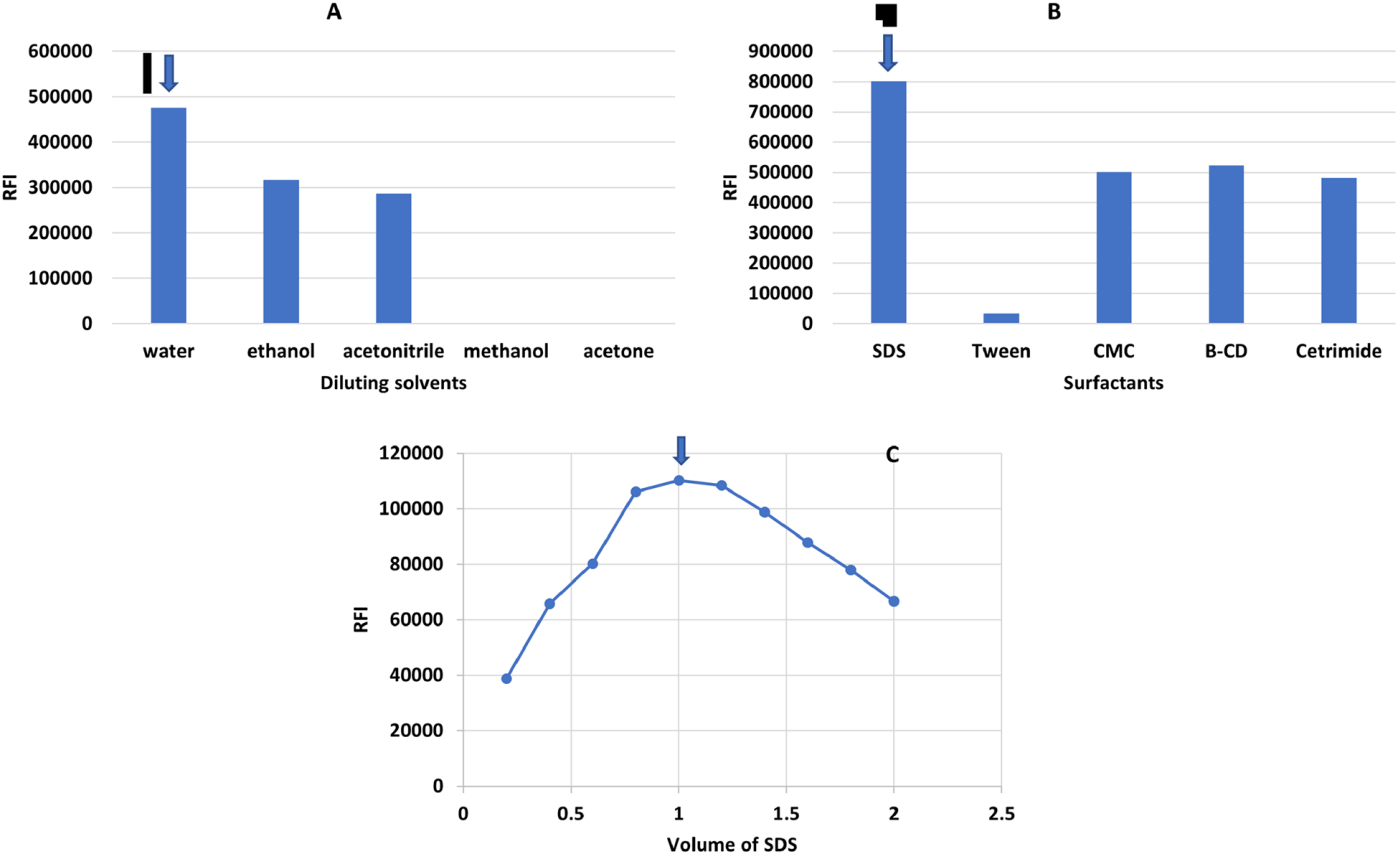
Optimization of the experimental conditions for determination of ORP: (**A**) effect of diluting solvent. (**B**) Effect of surfactant. (**C**) Effect of volume of 1.0 w/v % SDS.

**Figure 7:**
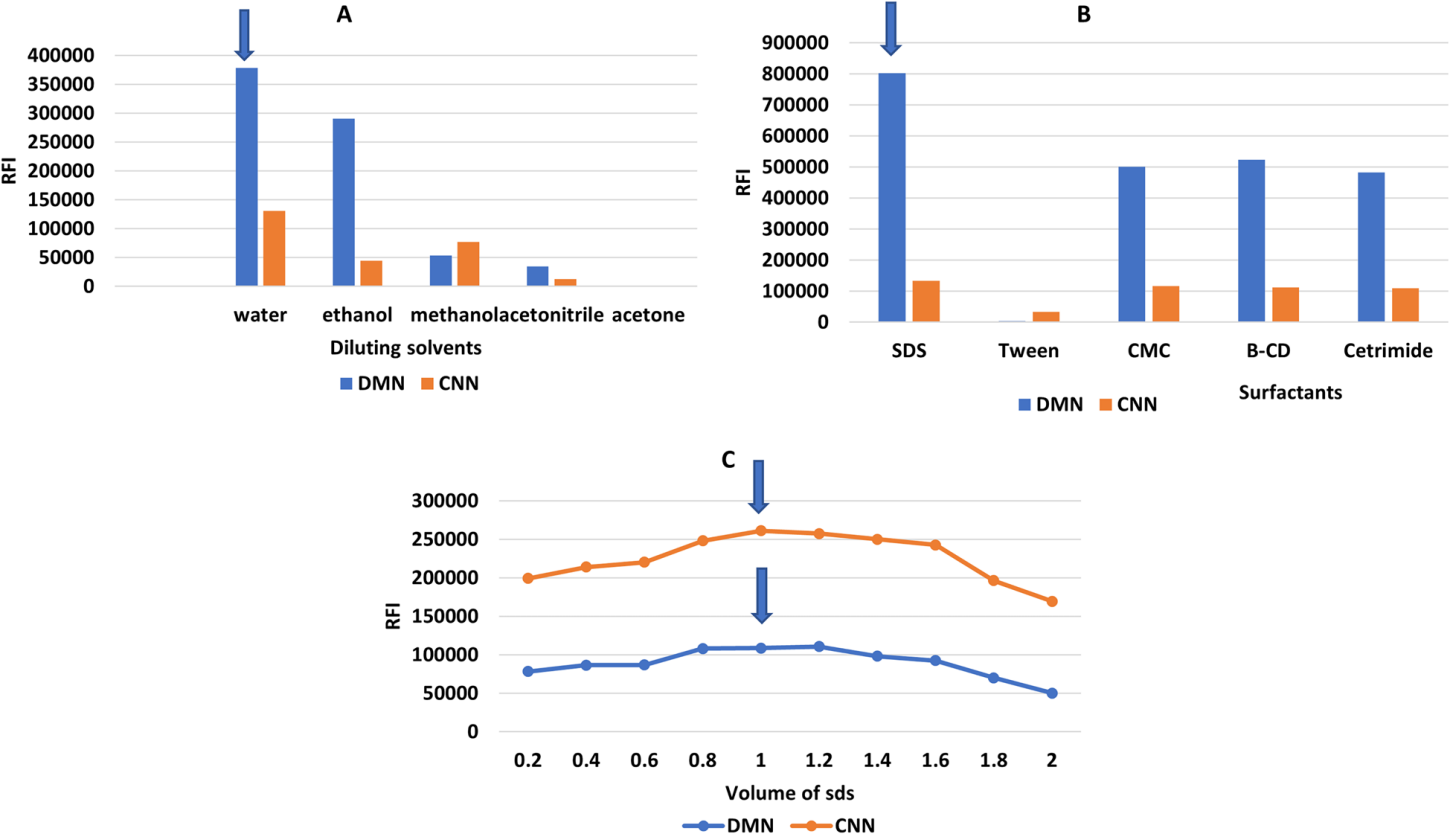
Optimization of synchronous conditions for determination of DMN and CNN: (**A**) effect of diluting solvent. (**B**) Effect of surfactant. (**C**) Effect of best volume of 1.0 w/v % SDS.

De-ionized water, acetonitrile, ethanol, methanol, n-propanol, and acetone are among the solvents that have been studied. In both techniques, de-ionized water was the most effective diluent as it greatly enhances the relative fluorescence intensity of dimenhydrinate, orphenadrine and cinnarizine compared to other diluting solvents; (Figs. 5, 6, 7—(A) this characteristic enhancing is most often observed with fluorophores that have large excited-state dipole moments, resulting in fluorescence spectral shifts to longer wavelengths in polar solvents. Therefore, it was chosen as the optimum solvent in all the studies. Furthermore, water is the greenest solvent when set against other solvents. Hence, its selection has a significant impact on the greenness of the developed methods.

#### Effect of pH

The conventional fluorescence of DMN (Method I) shows enhanced fluorescence intensity upon decreasing the pH of the analyte solution. Britton Robinson buffer (2.2–11.5), 0.1 M hydrochloric acid (HCl), 0.1 M sulfuric acid (H_2_SO_4_) and 0.1 M phosphoric acid (H_3_PO_4_) and sodium hydroxide (NaOH) were investigated. DMN is a weakly basic drug with a pKa 8.87. The maximum FI for DMN was observed in 0.1 M HCl. At this pH, DMN is fully protonated. Also, by comparing it to other acids, 0.1 M HCl was found to be the optimum acid as it produces the highest FI. Therefore, DMN analysis was performed in 0.1 M HCl (Fig. 5B). The fluorescence intensity was enhanced using a volume of 0.1 M HCl between 0.4 and 0.8 mL. Using volumes less than 0.4 mL, the acidity was not sufficient to achieve the highest fluorescence intensity while higher than 0.8 mL, the fluorescence intensity was decreased due to the heavy atom effect of the chlorine atom. The optimum volume of 0.1 M HCl was 0.6 mL (Fig. 5C) as it gave the highest quantitative fluorescence intensity and hence the highest fluorescence intensity.

In contrast to DMN, ORP exhibits a slight increase in the native fluorescence intensity in presence of 0.1 M hydrochloric acid. This effect is considered insignificant, therefore neither an acidic solution nor a buffer solution was utilized.

For the SFS of DMN and CNN (Method II), although DMN has high fluorescence intensity in 0.1 M HCl, the stability of CNN was highly affected by the acid medium due to its degradation^28^. Therefore, the simultaneous analysis of both analytes was performed without acidity.

#### Effect of surfactants

Three surfactants were investigated; cetrimide, SDS, CMC, and macromolecules such as: tween 80 and β cyclodextrin. For method I, SDS at a concentration of 1.0 w/v percent was chosen because it significantly increased the fluorescence intensity of DMN and ORP in a repeatable way Figs. 5D and 6B. SDS volumes of 0.2–2 mL were also investigated. 1.8 and 1.0 mL were found to be the best since they provided the highest FI for DMN and ORP, respectively (Figs. 5E and 6C). For method II, SDS was the best surfactant; 1 mL of 1.00% SDS could significantly enhance the SFS of DMN and CNN (Fig. 7B,C). The selected volume of 1%SDS produced high quantitative fluorescence intensities ± 0.2 mL; below the selected volumes lower FI were found and higher the selected ones producing constant FI. The role of SDS here can be explained in terms of viscosity because, at the studied SDS concentration, there are no micelles formed in the solution, but the used SDS increases the viscosity of the solution, and hence decreases the collisions between the molecules and hence decreases radiationless decay and loss of extra energy as heat leading to an increase in the fluorescence intensity.

#### Selection of the optimum Δλ

Varying the Δλ (20–200 nm) was performed to get the suitable Δλ at which the optimum sensitivity for both analytes was obtained. The sensitivity and resolution of the synchronous fluorescence were directly correlated with the optimal value of Δλ. For DMN and CNN, Δλ = 60 nm was the optimal wavelength because it produced well-defined spectra with less spectral interference—smaller or larger values of Δλ than the ideal one showed low SFI and poor separation.

The zero-order synchronous scans of DMN and CNN at Δλ = 60 nm produced overlapped spectra unsuitable enough to analyze both drugs simultaneously. Hence, mathematical manipulation of the zero-order synchronous fluorescence spectra was performed by applying different derivatives of high orders of the zero-order spectra of the studied analytes, such as first, second, third, and fourth-order derivatives. The first-order derivative of the synchronous fluorescent spectra succeeded in analyzing DMN and CNN with sufficient sensitivity and high selectivity, as illustrated in (Fig. 4B and D).

### Validation of the developed methods

Following ICH Q2 (R1), recommendations^27^, both techniques experienced testing to confirm that the validation requirements of linearity, range, selectivity, specificity, detection and quantitation limits, accuracy, and precision were met.

#### Linearity and range

Using the RFI or ^1^D values (FDSFS) in conjunction with the drug concentrations, linear ranges were determined from the calibration graphs. According to the fluorometric methodology (method I), the ranges for DMN and ORP were determined to be 0.1–1.0 µg/mL and 0.04–0.5 µg/mL, respectively. A good correlation between ^1^D values and drug concentrations in method II was accomplished in the range of 0.1–1.0 µg/mL for both DMN and CNN at 282 nm and 322 nm, respectively. The findings of the regression analysis and selected concentrations are shown in Tables 1 and 2.

**Table 1:** Analytical performance data for the determination of the studied drugs by the proposed methods.

Parameter	Method (I)		Method (II)	
Drug	DMN	ORP	DMN	CNN
	λex/λem	λex/λem		
Wavelength (nm)	222/286 nm	220/285 nm	282 nm	322 nm
Linearity range (µg/mL)	0.10–1.00	0.04–0.50	0.10–1.00	0.10–1.00
Intercept (*a*) × 10^3^	6.9	− 5.5	5.1	− 1.01
Slope (*b*) × 10^4^	13.31	22.51	15.07	8.82
Correlation coefficient (*r*)	0.9998	0.9999	0.9997	0.9999
S.D. of residuals (S_*y/x*_)	504.42	566.92	337.05	97.49
S.D. of intercept (S_*a*_)	120.99	321.46	80.84	26.38
S.D. of slope (S_*b*_)	646.734	1433.83	432.147	124.995
Percentage relative standard deviation, % RSD	1.11	0.72	1.30	0.77
Percentage relative error, % error	0.46	0.29	0.53	0.32
Limits of detection, LOD (ng/mL)	2.99	4.71	1.77	0.88
Limits of quantitation, LOQ (ng/mL)	9.08	14.29	5.36	2.65

**Table 2:** Application of the proposed methods for the determination of the studied drugs in pure forms.

Parameters	Method I						Method II						Comparison methods^9,14^					
	DMN at 286 nm			ORP at 285 nm			DMN at 282 nm			CNN at 322 nm			ORP		DMN		CNN	
	Amount taken µg/mL	Amount found µg/mL	%Found^a^	Amount taken µg/mL	Amount found µg/mL	%Found^a^	Amount taken µg/mL	Amount found µg/mL	%found^a^	Amount taken µg/mL	Amount found µg/mL	%found^a^	Amount taken µg/mL	% found^a^	Amount taken µg/mL	% found^a^	Amount taken µg/mL	% found^a^
	0.10	0.100	100.00	0.04	0.04	100.00	0.10	0.099	99.00	0.10	0.101	101.00	30.00	100.17	20.00	100.74	10.00	100.30
	0.20	0.199	99.50	0.10	0.1	100.00	0.20	0.197	98.50	0.20	0.201	100.50	40.00	100.78	40.00	99.48	20.00	99.57
	0.40	0.407	101.75	0.20	0.199	99.50	0.40	0.403	100.75	0.40	0.398	99.50	50.00	99.62	60.00	100.16	30.00	100.17
	0.60	0.595	99.17	0.30	0.304	101.33	0.60	0.597	99.50	0.60	0.594	99.00						
	0.80	0.790	98.75	0.40	0.397	99.25	0.80	0.815	101.88	0.80	0.806	100.75						
	1.00	1.008	100.80	0.50	0.501	100.20	1.00	0.989	98.90	1.00	0.999	99.90						
Mean			99.99	100.05					99.76			100.10		100.19		100.13	100.01	
± SD	1.11			0.72			1.30			0.76			0.58		0.63		0.39	
% RSD	1.11			0.75			1.30			0.76			0.58		0.63		0.39	
% Error	0.46			0.29			0.53			0.32			0.34		0.36		0.23	
*t*	0.19			0.29			0.46			0.19								
*F*	3.12			1.54			4.24			3.96								

The tabulated *t* and *F*- values 2.77 and 19 at *P* = 0.05, respectively^29^.

^a^Mean of three determinations.

#### Accuracy

Accuracy was examined by assessing specific concentrations of the investigated medications within the linear range and computing the % recoveries, as shown in Table 2. By determining the studied drugs in the pure and pharmaceutical dosage forms through the referred concentrations and comparing the results of the studied methods with the comparison methods^9,14^ by applying variance ratios *F-test* and student's *t-test*, accuracy was guaranteed.

#### Limits of detection and quantitation

The low values of detection and quantitation limits are illustrated in Table 1, ensuring the developed methods’ sensitivity. LOD is the lowest concentration that could be detected and calculated at 3.3 S_a_/b, while LOQ is the lowest concentration that could be quantified in terms of accuracy and precision and calculated at 10 S_a_/b.; where S_a_ means that the standard deviation of the intercept of the regression line is b, the slope of the calibration graph.

#### Precision

To guarantee the consistency and precision of the recommended methods, the following metrics were calculated: standard deviation (SD), mean, relative standard deviation (RSD), and relative percentage error (% Error). The intraday precision (repeatability) and interday precision were evaluated by assessing three different concentrations and measuring them three times on the same day or over three days, respectively (Table 3). The results showed that the approaches were highly precise (RSD < 2%).

**Table 3:** Precision data for the estimation of studied drugs by the proposed methods.

Parameters		Method (I)						Method (II)					
		DMN (µg/mL)			ORP (µg/mL)			DMN (µg/mL)			CNN (µg/mL)		
		0.10	0.20	0.40	0.10	0.20	0.40	0.10	0.20	0.40	0.10	0.20	0.40
Intra-day	Mean	100.00	99.83	99.92	100.00	100.17	100.00	99.67	99.83	99.92	99.66	100.00	100.00
	± SD	1.00	0.76	0.38	0.87	1.44	0.43	1.15	0.29	0.14	0.58	0.50	0.50
	% RSD	1.00	0.77	0.38	0.87	1.44	0.43	1.15	0.29	0.14	0.58	0.50	0.50
	% Error	0.58	0.44	0.22	0.5	0.83	0.25	0.67	0.17	0.08	0.33	0.29	0.29
Inter-day	Mean	100.33	100.00	100.08	100.33	100.00	99.92	100.00	100.00	100.00	100.00	99.83	100.00
	± SD	1.15	1.50	1.63	1.15	0.86	1.53	1.00	1.32	0.66	1.00	1.89	1.00
	% RSD	1.15	1.50	1.63	1.15	0.86	1.53	1.00	1.32	0.66	1.00	1.89	1.00
	% Error	0.67	0.87	0.94	0.67	0.50	0.88	0.58	0.76	0.38	0.58	1.09	0.58

Each result is the average of three separate determinations.

#### Robustness

The robustness of the suggested techniques was verified by assessing the effect of small deliberate changes in variable parameters involved, such as in method I; the variations in pH (1.3 ± 0.2) and volume of the acid (0.6 mL ± 0.2 mL) and surfactant 1% w/v SDS (1.8 mL ± 0.2 mL) for DMN, (1 mL ± 0.2 mL) for ORP. Method II (1 mL ± 0.2 mL) of 1% w/v SDS for DMN and CNN was performed. It was found that these small, intended changes do not affect the RFI or the D^1^ amplitudes, respectively for both methods.

#### Selectivity

The selectivity was evaluated by testing for excipient interference in the pharmaceutical formulations using both methods. Talc, magnesium stearate, or lactose did not cause any interference. Additionally, the FDSFS could quantify DMN and CNN independently at their zero crossings. The obtained % recoveries of the two drugs in their pharmaceutical preparations range from (99.48–100.74) and (99.00–101.0) with RSD (< 2%) for DMN and ORP (Method I) and for DMN and CNN for (Method II), respectively, indicating the selectivity of the results.

### Applications

#### Analysis of DMN/CNN synthetic mixtures:

**Figure 8:**
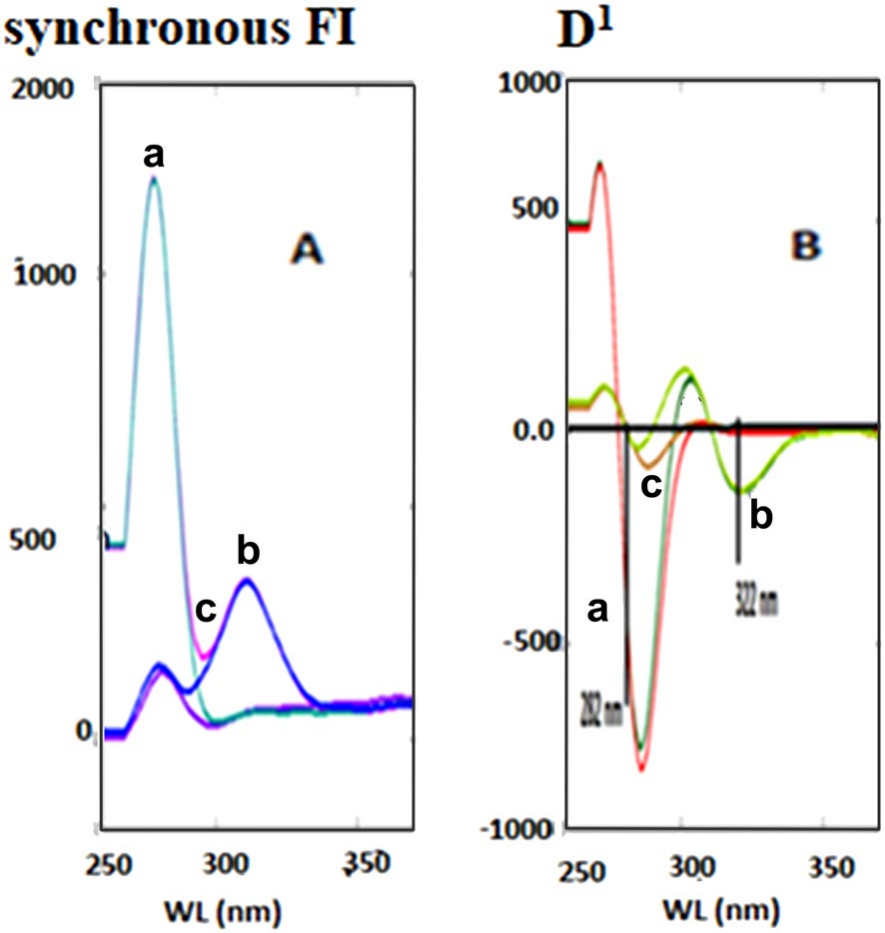
(**A**) synchronous fluorescence spectroscopy (SFS) conditions at Δλ = 60 nm of (a; 0.8 µg/mL DMN, b; 0.4 µg/mL CNN and c; synthetic mixture of DMN and CNN, respectively). **(B**) first derivative synchronous fluorescence spectroscopy (FDSFS) at Δλ = 60 nm of (a; 0.8 µg/mL DMN, b; 0.4 µg/mL CNN and c; synthetic mixture of DMN and CNN, respectively).

The proposed first derivative synchronous method was utilized to analyze the two drugs in their synthetic mixture. Other ratios besides their pharmaceutical ratio of 2:1 w/w (DMN: CNN) were studied. Table S1 showed acceptable % recoveries for both drugs. Figure 8A,B indicates the SFS and FDSFS of the two analytes in their synthetic mixture according to their pharmaceutical ratio.

#### Analysis of DMN/ORP/CNN in single formulations and DMN/CNN in combined tablets

First of all, DMN, ORP, and CNN were analyzed in their single dosage forms; tablets (Dramanex^®^), ampoules (Norflex^®^), and capsules (Cinnarizine^®^), respectively while DMN and CNN were also analyzed in their prepared combined tablet. The results were obtained by applying the conventional fluorometric technique for DMN and ORP and FDSFS for DMN and CNN; then, the results were compared with those of the comparison methods^9,14^. Since the tabulated values of *t*- and *F-*tests^29^ were more significant than the calculated values, the accuracy and precision were confirmed. Moreover, no characteristic interferences from additives were observed, ensuring the high specificity of the developed methods, as shown in Tables 4 and 5.

**Table 4:** Determination of the studied drugs in pharmaceutical preparations using the proposed methods.

Compound	Proposed method			Comparison methods^9,14^	
	Amount taken (μg/mL)	Amount found (μg/mL)	% Found	Amount taken (μg/mL)	% Found
Method I					
Dramanex^®^ tablets DMN (50.0 mg)/tablet	0.20	0.199	99.50	20.00	100.74
	0.40	0.402	100.50	40.00	99.48
	0.80	0.799	99.88	60.00	100.016
${\overline{\text{x}}}$ ± SD			99.96 ± 0.51		100.13 ± 0.63
* t*			0.25		
* F*			0.63		
Norflex® ampoules ORP (30 mg/mL)/ampoule	0.20	0.198	99.00	30.00	100.17
	0.30	0.304	101.00	40.00	100.78
	0.40	0.398	99.50	50.00	99.62
${\overline{\text{x}}}$ ± SD			99.94 ± 1.23		100.19 ± 0.58
* t*			0.52		
* F*			3.21		
Method II					
Dramanex^®^ tablets DMN (50.0 mg)/tablet	0.20	0.199	99.50	20.00	100.74
	0.40	0.401	100.25	40.00	99.48
	0.80	0.800	100.00	60.00	100.016
${\overline{\text{x}}}$ ± SD			99.92 ± 0.38		100.13 ± 0.63
* t*			0.38		
* F*			0.36		
Cinnarizine^®^ capsules CNN (75.0 mg)/capsule	0.20	0.198	99.00	10.00	100.30
	0.40	0.404	101.00	20.00	99.57
	0.60	0.598	99.67	30.00	100.17
${\overline{\text{x}}}$ ± SD			99.89 ± 1.02		100.01 ± 0.39
* t*			0.19		
* F*			6.83		

The tabulated *t* and *F*-values 2.77 and 19 at *P* = 0.05, respectively^29^.

^a^Mean of three determinations.

**Table 5:** Application of the proposed methods to determine DMN and CNN in prepared combined tablets.

Parameter	Proposed method			Comparison methods^9,14^		
	Amount taken (µg/mL)	Amount found (µg/mL)	Percentage found^a^	Amount taken (µg/mL)	Amount found (µg/mL)	Percentage found^a^
DMN	0.20	0.202	101.00	20.00	20.147	100.74
	0.40	0.397	99.25	40.00	39.791	99.32
	0.80	0.801	100.13	60.00	60.095	100.16
Mean			100.13			100.13
± S.D			0.88			0.63
% RSD			0.88			0.63
% Error			0.51			0.36
*t*	0.08					
*F*	1.92					
CNN	0.10	0.099	99.00	10.00	10.03	100.30
	0.20	0.201	100.50	20.00	19.914	99.57
	0.40	0.400	100.00	30.00	30.05	100.17
Mean			99.83			100.01
± S.D			0.76			0.39
% RSD			0.76			0.39
% Error			0.44			0.23
*t*	0.36					
*F*	3.85					

The tabulated* t* and *F*- values 2.77 and 19 at *P* = 0.05, respectively^29^.

^a^Mean of three determinations.

#### Assessment of the green property

Given the extensive usage of organic solvents in analytical processes, becoming green can be difficult. Any analytical procedure can be made greener by reducing the use of these solvents or by substituting greener ones. The eco-friendliness of these methods was assessed in two different ways. The Green Analytical Procedure Index (GAPI), is a more contemporary tool for gauging greenness^30^. It adheres to every procedure phase, from sample collecting to trash treatment. It has 15 items to be evaluated using one of three degrees of color (green, yellow, or red) and provides a detailed evaluation for each stage in the analytical technique. Table 6 displays the green profiles for the suggested spectrofluorometric methods using the GAPI tool. Under normal circumstances, DMN, ORP, and CNN must be stored in aluminum foil and a refrigerator; hence the fourth parameter was highlighted in yellow in both approaches. The fifth parameter is highlighted in yellow since both methods involved sample preparation and filtration. The two pictograms (10, 11) pertaining to the reagents and solvents were yellow shaded for DMN due to the use of some hazardous chemicals like hydrochloric acid, SDS, even though their usage was by small volume; as a result, their national fire protection association (NFPA) health hazard rating exceeds two; however, it shaded green for ORP and method II as their NFPA rating is 2. Some approaches may be oppressed by GAPI evaluation. The amount of waste was between 1 and 10 mL; thus, it was tinted yellow in field no. 14, while in field No. 15, all techniques had red coloring because there was no waste treatment.

**Table 6:** Results for the greenness evaluation of the developed conventional method by GAPI and analytical eco scale green chemistry tools (method I and II).

1—Green analytical procedure index (GAPI)					
Method I DMN	Method I ORP	Method II			
					

2—Analytical eco scale score					
Reagents/instruments					
Reagent, volume (mL)	No of pictograms	Word sign	Penalty points		
Method I DMN					
1% SDS, 1.8 mL	1	Warning	1		
0.1M hydrochloric acid, 0.6mL	1	Danger	2		
Water			0		
Method I ORP					
1% SDS, 1 mL	1	Warning	1		
Water			0		
Method II					
1% SDS, 1 mL	1	Warning	1		
Water			0		
Item for all methods			Penalty points		
Spectrofluorometer	< 0.1 k w h per sample		0		
Waste	No treatment		3		
Occupational hazards	Analytical process hermitization		0		
Total penalty points			⅀ 6	⅀ 4	⅀ 4
Analytical eco scale score for both methods			100–6 = 94	100–4 = 96	100–4 = 96
			Method I DMN	Method I ORP	Method II

Analytical eco scale is another quantitative assessment tool published by Van-Akan et al.^31^. Depending on the number of penalty points, grading the method's greenness. The number of pictograms and signal words included in "The Globally Harmonized System of Classification and Labeling of Chemicals" (GHS) and the safety label data sheet for each chemical or solvent is recorded as penalty points, which are then deducted from 100. As demonstrated in Table 6, superior green methods received 75 or more points, while good green received 50 points or more. The synchronous technique received 96, whereas the conventional method received 94 and 96 for DMN and ORP, respectively. Regarding the analytical eco-scale criteria, both approaches excel. The National Fire Protection Association determined the penalty points (NFPA)^32^.

This was over-assessed using the analytical greenness calculator and the AGREE metric^33^. The AGREE metric is a novel assessment method that depends on the 12 principles of green analytical chemistry (GAC), abbreviated as SIGNIFICANCE. It appears to be a clock watch. The parameters were numbered from 1 to 12, each assigned a score between 0 and 1, with the final score added in the middle. This model is green, with shades of orange, yellow, and red and lighter and deeper green. When the score is one or nearly one, green shading appears; it changes to yellow or red shades when it is less than one. The proposed spectrofluorimetric methods are evaluated for their greenness using the AGREE metric, as shown in Table 7. Method I for DMN has a yellow zone due to the use of hydrochloric acid, which is corrosive, and one red zone due to the high number of samples examined per hour. The safety label data sheet shows two yellow zones due to the composition of reagents and waste disposal. This application can be downloaded for free at https://mostwiedzy.pl/AGREE.

**Table 7:** Assessment of greenness of the proposed spectrofluorometric methods using AGREE metric.

Method I DMN		Method I ORP		Method II	
AGREE plots					
		
Criteria scores					
1. Direct analytical techniques should be applied to avoid sample reatment	0.7	1. Direct analytical techniques should be applied to avoid sample treatment	0.7	1. Direct analytical techniques should be applied to avoid sample treatment	0.7
2. Minimal sample size and minimal number of samples are goals	0.66	2. Minimal sample size and minimal number of samples are goals	0.66	2. Minimal sample size and minimal number of samples are goals	0.66
3. If possible, measurements should be performed in situ	0.66	3. If possible, measurements should be performed in situ	0.66	3. If possible, measurements should be performed in situ	0.66
4. Integration of analytical processes and operations saves energy andreduces the use of reagents	1.0	4. Integration of analytical processes and operations saves energy and reduces the use of reagents	1.0	4. Integration of analytical processes and operations saves energy and reduces the use of reagents	1.0
5. Automated and miniaturized methods should be selected	0.75	5. Automated and miniaturized methods should be selected	0.75	5. Automated and miniaturized methods should be selected	0.75
6. Derivatization should be avoided	1.0	6. Derivatization should be avoided	1.0	6. Derivatization should be avoided	1.0
7. Generation of a large volume of analytical waste should be avoided, and proper management of analytical waste should be provided	0.67	7. Generation of a large volume of analytical waste should be avoided, and proper management of analytical waste should be provided	0.67	7. Generation of a large volume of analytical waste should be avoided, and proper management of analytical waste should be provided	0.67
8. Multi-analyte or multi-parameter methods are preferred versus methods using one analyte at a time	0.0	8. Multi-analyte or multi-parameter methods are preferred versus methodsusing one analyte at a time	0.0	8. Multi-analyte or multi-parameter methods are preferred versus methods using one analyte at a time	0.0
9. The use of energy should be minimized	0.71	9. The use of energy should be minimized	0.71	9. The use of energy should be minimized	0.71
10. Reagents obtained from renewable sources should be preferred	0.5	10. Reagents obtained from renewable sources should be preferred	1.0	10. Reagents obtained from renewable sources should be preferred	1.0
11. Toxic reagents should be eliminated or replaced	1.0	11. Toxic reagents should be eliminated or replaced	1.0	1. Toxic reagents should be eliminated or replaced	1.0
12. Operator's safety should be increased	0.6	12. Operator's safety should be increased	1.0	12. Operator's safety should be increased	0.8

The created methodologies are compatible with the three green analytical chemistry tools, which explains why they are simple, sensitive, quick, and eco-friendly.

## Conclusion

A green, simple, and highly sensitive conventional fluorometric method is established to quantify ORP or DMN in pharmaceutical dosage forms. Moreover, a first derivative synchronous fluorescence spectroscopy is used as simple, selective, and green technique to determine DMN and CNN in pure forms and in their pharmaceuticals. Owing to the simplicity and sensitivity of the proposed methods, they can be an excellent alternative to other sophisticated techniques in quality control units. This work, besides being simple for application in different quality control units on different dosage forms as investigated through this work, is also highly sensitive as identified through the linearity range for each drug.

### Supplementary Information

Supplementary Table S1.

## Data Availability

All data generated or analyzed during this study are included in this published article and its supplementary information files.
