# Supplementary material for: Spectrofluorometric determination of orphenadrine, dimenhydrinate, and cinnarizine using direct and synchronous techniques with greenness assessment
Source: Sci Rep. 2023 Aug 20;13:13549. doi: 10.1038/s41598-023-40559-x (PMC10440341; doi:10.1038/s41598-023-40559-x)
Supplement: Supplementary file 1 — Supplementary Table S1. [file 41598_2023_40559_MOESM1_ESM.docx]

**Spectrofluorometric determination of orphenadrine, dimenhydrinate, and cinnarizine using direct and synchronous techniques with greenness assessment**

**Rana Ghonim ^a, b*^, Manar M. Tolba ^a^, Fawzia Ibrahim ^a^, Mohamed I. El-Awady ^a, b^**

1. *Department of Pharmaceutical Analytical Chemistry, Faculty of Pharmacy, Mansoura University, Mansoura 35516, Egypt.*
2. *Department of Pharmaceutical Chemistry, Faculty of Pharmacy, Delta University for Science and Technology, International Coastal Road, Gamasa 11152, Egypt.*

**Supporting information:**

1. Assay results for the determination of DMN and CNN in synthetic mixtures using proposed Method (II).

**Table S1:** Assay results for the determination of DMN and CNN in synthetic mixtures using proposed Method (II).

| Percentage found^a^ | | Amount found  (µg/mL) | | Amount taken  (µg/mL) | | Ratio | Mix. No |
| --- | --- | --- | --- | --- | --- | --- | --- |
| DMN | **CNN** | **DMN** | **CNN** | **DMN** | **CNN** |  |  |
| 100.00 | 98.50 | 0.400 | 0.394 | 0.40 | 0.40 | 1:1 | 1 |
| 99.38 | 100.25 | 0.795 | 0.802 | 0.80 | 0.80 | 1:1 | 2 |
| 100.00 | 101.00 | 0.400 | 0.202 | 0.40 | 0.20 | 1:2 | 3 |
| 101.17 | 101.00 | 0.607 | 0.303 | 0.60 | 0.30 | 1:2 | 4 |
| 99.38 | 98.50 | 0.795 | 0.394 | 0.80 | 0.40 | 1:2 | 5 |
| 99.00 | 101.00 | 0.099 | 0.202 | 0.10 | 0.20 | 2:1 | 6 |
| 99.50 | 98.50 | 0.199 | 0.394 | 0.20 | 0.40 | 2:1 | 7 |
| 100.00 | 100.25 | 0.400 | 0.802 | 0.40 | 0.80 | 2:1 | 8 |
| 99.81 | 100.19 |  | | | |  | **Mean** |
| 0.84 | 1.18 |  |  |  |  |  | **± S.D.** |
| 0.84 | 1.18 |  |  |  |  |  | **%RSD** |
| 0.38 | 0.59 |  |  |  |  |  | **%Error** |

^a^ Mean of three determinations
